# Acceptability of Vaginal Human Papillomavirus Self-Collection Among Colposcopy Clinic Attendees

**DOI:** 10.1089/whr.2025.0006

**Published:** 2025-04-14

**Authors:** Kathy L. MacLaughlin, Kristin C. Cole, Julie A. Maxson, Jainnee McCann, Xuan Zhu, Robert M. Jacobson, Matthew R. Meunier, Margaret E. Long

**Affiliations:** ^1^ Department of Family Medicine, Mayo Clinic, Rochester, Minnesota, USA.; ^2^ Department of Quantitative Health Sciences, Mayo Clinic, Rochester, Minnesota, USA.; ^3^ Department of Obstetrics & Gynecology, Mayo Clinic, Rochester, Minnesota, USA.; ^4^ Kern Center for the Science of Health Care Delivery, Mayo Clinic, Rochester, Minnesota, USA.; ^5^ Department of Pediatric and Adolescent Medicine, Mayo Clinic, Rochester, Minnesota, USA.

**Keywords:** human papillomavirus DNA tests, uterine cervical neoplasms, early detection of cancer, patient preference

## Abstract

**Background:**

Cervical cancer screening with a self-collected vaginal specimen for human papillomavirus (HPV) testing was approved by the U.S. Food and Drug Administration in May 2024, offering a potential solution to declining screening rates.

**Objective:**

We aimed to assess acceptability of clinic-based vaginal specimen self-collection for HPV testing and to evaluate associations between participants’ sociodemographics and their likelihood of choosing self-collection for future screening and the overall acceptability of using the Evalyn® brush device. We also evaluated associations between specific acceptability constructs and reported likelihood to use the device in the future and overall acceptability.

**Methods:**

Following self-collection of a vaginal specimen, participants completed an electronic survey that measured constructs from the Theoretical Framework of Acceptability. Associations were assessed using logistic regressions. The study was conducted at a colposcopy clinic in the Gynecology department of a midwestern academic medical center in the United States from November 2022 through July 2023.

**Results:**

Participants (*n* = 81) reported high likelihood (98% likely or very likely) of choosing in-home self-collection for future screening and unanimity on overall acceptability (100% acceptable or completely acceptable) of using the device. More affirmative responses to measures on instruction understandability, ease of device use, understanding the device is used for cervical cancer screening, and perceiving self-collection improves screening rates were associated with a higher likelihood to choose self-collection for future screenings and overall acceptability of the device (all *p* values <0.05).

**Conclusion:**

Vaginal specimen self-collection for HPV testing was well-received in the studied population, with high acceptability and likelihood of uptake. Implementation efforts should provide user-friendly instructions and emphasize the benefits of self-collection for cervical cancer screening, particularly among people less likely to engage with clinician-collected speculum-based screening.

## Introduction

Although cervical cancer is preventable through human papillomavirus (HPV) vaccination in adolescence, regular screening in adulthood and treatment of identified precancers, it remains the fourth most common cancer among women worldwide.^1^ In the United States, the American Cancer Society estimates almost 14,000 new cases and just over 4,000 deaths from cervical cancer in 2024.^2^ Concerning declines in U.S. cervical cancer screening rates over the past two decades have been observed in the National Health Interview Survey, with 86.5% of screening-eligible women in 2000 reporting up-to-date screening status but only 72.4% in 2021, with even lower screening rates associated with lower income, lower education level, and among non-Hispanic Black and Hispanic women.^3^ Self-collection of a vaginal specimen for HPV testing for cervical cancer screening is already offered in many countries worldwide as a way to reach those who may not otherwise be screened.^4^ In May 2024, the U.S. Food and Drug Administration (FDA) approved HPV test self-collection for use in a health care setting with the BD Onclarity™ HPV Assay or Roche cobas® HPV test lab platforms, paving the way for expanded access to screening in the United States.^5^

The introduction and expansion of the HPV self-collection screening option is based on the high percentage agreement between the HPV results for self-collected vaginal specimens and clinician-collected cervical specimens as well as the comparable sensitivity of self-collected vaginal HPV testing to detect cervical precancer when compared with clinician-collected cervical HPV testing, using PCR-based HPV assays.^6,7^ This novel screening option has the potential to address barriers to traditional cervical cancer screening with a clinician-conducted pelvic and speculum exam and may improve screening rates, especially among those who have been historically unscreened or underscreened. Barriers to speculum-exam-based cervical cancer screening that may be mitigated by self-collection include physical disabilities,^8^ obesity,^9^ a history of sexual abuse,^10^ sexual or gender minority status,^11,12^ mental health disabilities,^13^ and cultural or religious beliefs.^14^

Studies in the United States have observed acceptance of HPV test self-collection across diverse groups in low-resource settings including Appalachian Kentucky,^15^ Somali immigrants in Minnesota,^16^ Latina and Haitian women in Miami,^17^ Black women in the Mississippi Delta,^18^ and among low-income underscreened women in North Carolina.^19^ These studies have evaluated self-collection in various settings including homes, clinics, and a church. However, less is known about the acceptance of self-collection among those in higher resource settings. Although higher screening rates are often anticipated in resource-rich settings, low screening rates in line with 2021 National Health Interview Survey data were reported in a midwestern county with access to community and tertiary care centers, where our study was conducted.^20^

For this survey study, our primary aim was to assess acceptability of completing a clinic-based vaginal specimen self-collection for HPV testing from the participant perspective in a higher resource setting. The survey incorporated acceptability constructs from a generic questionnaire based on the Theoretical Framework of Acceptability (TFA), a resource intended to be adapted to assess acceptability of health care interventions.^21^ Additionally, we aimed to evaluate the associations between participant sociodemographic factors and their future likelihood of choosing a home-based self-collection option for cervical cancer screening along with overall acceptability of using the HPV self-collection device. We also evaluated associations between survey responses to specific acceptability constructs and reported likelihood to use the device in the future along with overall acceptability.

## Materials and Methods

### Study population

We conducted the survey study from November 2022 to July 2023 at the colposcopy clinic in the Gynecology department of a midwestern academic medical center, primarily serving local and regional patients. The survey was embedded within an HPV self-collection device validation study using the Evalyn® brush prior to the device’s FDA approval (Rovers Medical Devices B.V., Oss, the Netherlands). As of May 2024, the Evalyn® brush is an FDA-approved self-collection device that may be used with the Roche cobas HPV test lab platform.^5^ Inclusion criteria for participants included those aged 25–65 years old scheduled for an appointment that would include a speculum exam. Exclusion criteria included history of total hysterectomy, loop electrosurgical excision procedure scheduled for day of the upcoming appointment, pregnant or within 3 months postpartum, abnormal vaginal discharge or moderate to heavy vaginal bleeding on day of the appointment, or use of vaginal products within 2 days of appointment (other than water-based lubricants). The sample size was determined through power calculations for validating the HPV self-collection device, incorporating estimated prevalence of HPV infection among colposcopy clinic patients and a set target sensitivity level. This resulted in a minimum required sample size of 74.

The study was reviewed and approved by our academic medical center’s institutional review board.

### Survey design and measures

In designing our cross-sectional study, we adapted an existing generic survey tool to assess participant perspectives on the HPV test self-collection with the Evalyn® brush, based on constructs from the TFA, first proposed by Sekhon et al. in 2017.^22^ The TFA provides a rigorous structure for evaluating the acceptability of health care interventions, including constructs such as affective attitude, burden, ethicality, perceived effectiveness, intervention coherence, self-efficacy, and opportunity costs, along with a recommendation to include a general acceptability question.^21^ In our survey, we assessed *affective attitude* by asking about participants’ comfort level before collecting the vaginal specimen. We assessed *burden* by asking how understandable the written instructions were for using the self-collection device and the ease of using the device. To assess *self-efficacy*, we included a question on participants’ confidence in correctly collecting the sample. To assess *intervention coherence*, we asked if the participant understood that using the self-collection device would allow for cervical cancer screening by HPV testing. To assess *perceived effectiveness*, we asked if allowing patients to collect their own samples could improve screening rates. We framed *opportunity costs* as participants’ likelihood to choose home-based self-collection for future screening if offered by their health care provider. Finally, we measured *general acceptability* by asking how acceptable it was overall to collect the sample using the self-collection device. For each question, we used a 4-point Likert scale, omitting the middle “no opinion” option from the original 5-point Likert scale to encourage a clearer positive or negative response and to reduce ambiguity. In presenting the TFA, Sekhon et al. acknowledged that not all constructs are applicable to every health care intervention; therefore, we did not include the construct of *ethicality* (fairness or moral consequences to intervention) in our survey.^21^ The survey was pilot-tested with 10 females, over half of whom were not medically trained. Based on their feedback, the survey language was simplified and questions were shortened.

Sociodemographic characteristics of participants were obtained from the electronic medical record, including age, race/ethnicity, body mass index (BMI), and rurality of home address classified by the Rural-Urban Continuum Codes. Social drivers of health including self-reported physical activity level, stress concern, social connectedness, risk for intimate partner violence, financial strain, food insecurity, transportation needs, alcohol misuse, and smoking status were also obtained from the electronic medical record.

### Survey administration

Enrolled participants were roomed in the clinic per usual protocol after checking in. Next, the study coordinator provided each participant with an Evalyn® brush self-collection device and printed self-collection instructions. Participants remained alone in the exam room, collecting their vaginal specimen in private. After the participant completed self-collection, the study coordinator returned to the exam room and gave the participant an iPad to complete the electronic survey. Subsequently, the clinician scheduled to meet with the participant conducted a speculum exam, collecting a cervical specimen for HPV testing as part of the study’s validation component before performing the colposcopy.

### Statistical analysis

We reported descriptive statistics for participant sociodemographic characteristics and the survey-based acceptability constructs, including frequencies and percentages. We used logistic regression to examine the associations between sociodemographics and the likelihood of choosing self-collection for cervical cancer screening at home in the future (being “very likely” as compared with being “likely”) as well as the overall acceptability of using the HPV self-collection device (“completely acceptable” as compared with “acceptable”). Similarly, we used logistic regression to examine the associations between survey responses to specific acceptability constructs, including affective attitude (comfort level precollection), burden (ease of use of device), self-efficacy (confidence in collection), intervention coherence (understanding that the device could be used for screening), and perceived effectiveness (agreeing the self-collection could improve screening rates), and the likelihood to choose self-collection in the future and the overall acceptability of using the device.

Associations were summarized using odds ratios (ORs) and 95% confidence intervals (CIs). Missing data were excluded from that respective comparison (*i.e.,* no imputation for missing data was done). All tests were two-sided, and *p* values ≤0.05 were considered statistically significant. All analyses were performed using SAS version 9.4 (SAS Institute Inc., Cary, NC, USA).

## Results

### Descriptive survey results

Our survey sample (*n* = 81) consisted mostly of White women (92%), most of whom were never smokers (65%), living in a metro area (85%), with a BMI in the overweight or obese range (61%) and with a mean age of 42. Although 19% reported uncomfortable or very uncomfortable before collecting the sample, all participants (100%) found the written instructions easy or very easy to understand, and 93% reported being confident (52%) or very confident (41%) that they had collected the sample correctly. Additionally, 99% agreed or strongly agreed that the Evalyn® brush could be used for cervical cancer screening by testing for HPV, and 99% agreed or strongly agreed that allowing self-collection would improve cervical cancer screening rates. Support for choosing self-collection for future home-based screening was high with 25% indicating they were likely and 73% very likely to choose this option if offered by their provider. Overall acceptability of using the self-collection device was also high, with 19% finding it acceptable and 81% finding it completely acceptable (Table 1).

**Table 1.** Survey Results of HPV Self-Collection Study Participants (Acceptability Construct Domains)

	Total (*N* = 81)
“What was your comfort level before you collected the sample?” (*affective attitude*)^a^	
Very uncomfortable	10 (12%)
Uncomfortable	5 (6%)
Comfortable	30 (38%)
Very Comfortable	35 (44%)
“Understanding the written instructions for using the Evalyn® brush was _______.” (*burden*)	
Very difficult	0 (0%)
Difficult	0 (0%)
Easy	27 (33%)
Very easy	54 (67%)
“Using the Evalyn® brush to collect a sample by yourself for potential cervical cancer screening was _______.” (*burden*)	
Very difficult	0 (0%)
Difficult	1 (1%)
Easy	22 (27%)
Very easy	58 (72%)
“How confident are you that you collected the sample correctly?” (*self-efficacy*)	
Very unconfident	0 (0%)
Unconfident	6 (7%)
Confident	42 (52%)
Very confident	33 (41%)
“I understand that collecting the sample using the Evalyn® brush would allow for potential cervical cancer screening by HPV testing.” (*intervention coherence*)^a^	
Strongly disagree	1 (1%)
Disagree	0 (0%)
Agree	26 (33%)
Strongly agree	53 (66%)
“Allowing patients to collect their own sample will improve potential cervical cancer screening rates.” (*perceived effectiveness*)	
Strongly disagree	1 (1%)
Disagree	0 (0%)
Agree	25 (31%)
Strongly agree	55 (68%)
“In the future, if your healthcare provider offered the option to use the Evalyn® brush for potential cervical cancer screening at home, how likely would you be to choose that option?” (*opportunity costs*)^b^	
Very unlikely	0 (0%)
Unlikely	1 (1%)
Likely	20 (25%)
Very likely	58 (73%)
“Overall, how acceptable was it for you to collect the sample using the Evalyn® brush?” (*general acceptability*)	
Completely unacceptable	0 (0%)
Unacceptable	0 (0%)
Acceptable	15 (19%)
Completely acceptable	66 (81%)

^a^ Missing survey response in 1 participant.

^b^ Missing survey response in 2 participants.

HPV, human papillomavirus.

#### Association between sociodemographic factors and likelihood of choosing home-based self-collection for screening

No significant associations were observed between the sociodemographic variables and the reported strength of likelihood (very likely compared with likely) of choosing self-collection for future screenings (Table 2).

**Table 2.** Association Between Sociodemographic Factors and Likelihood of Choosing Home-Based Self-Collection for Screening Among HPV Self-Collection Study Participants

	Total (*N* = 78)	Very likely (*N* = 58)	Likely (*N* = 20)	OR (95% CI)	*p*-Value
Age, mean (SD)	42.3 (12.4)	42.3 (12.4)	42.4 (12.5)	1.00 (0.96–1.04)	0.98
Race/ethnicity					
White	72 (92%)	53 (91%)	19 (95%)	Reference	
Hispanic or Latino	4 (5%)	4 (7%)	0 (0%)	3.28 (0.12–89.66)	0.48
Other/choose not to disclose	2 (3%)	1 (2%)	1 (5%)	0.37 (0.02–6.12)	0.48
Body mass index^a^					
Normal/underweight	27 (39%)	19 (38%)	8 (42%)	Reference	
Overweight	18 (26%)	14 (28%)	4 (21%)	1.47 (0.37–5.88)	0.58
Obese	24 (35%)	17 (34%)	7 (37%)	1.02 (0.31–3.42)	0.97
Rural-Urban Continuum Codes					
Metro	66 (85%)	48 (83%)	18 (90%)	Reference	
Nonmetro	12 (15%)	10 (17%)	2 (10%)	1.88 (0.37–9.40)	0.44
Physical activity^b^					
Sufficiently active	29 (38%)	19 (33%)	10 (50%)	Reference	
Insufficiently active	42 (55%)	33 (58%)	9 (45%)	1.93 (0.67–5.59)	0.23
Inactive	6 (8%)	5 (9%)	1 (5%)	2.63 (0.27–25.72)	0.41
Stress concern present^c^	32 (42%)	25 (45%)	7 (35%)	1.50 (0.52–4.32)	0.45
Social connections^d^					
Socially isolated	23 (32%)	20 (38%)	3 (15%)	Reference	
Moderately isolated	18 (25%)	12 (23%)	6 (30%)	0.30 (0.06–1.43)	0.13
Moderately integrated	19 (26%)	12 (23%)	7 (35%)	0.26 (0.06–1.19)	0.082
Socially integrated	13 (18%)	9 (17%)	4 (20%)	0.34 (0.06–1.83)	0.21
At risk of intimate partner violence^e^	5 (7%)	5 (9%)	0 (0%)	4.17 (0.17–100.00)	0.38
Financial resource strain^c^					
Low risk	50 (66%)	35 (63%)	15 (75%)	Reference	
Medium risk	10 (13%)	8 (14%)	2 (10%)	1.71 (0.33–9.05)	0.53
High risk	16 (21%)	13 (23%)	3 (15%)	1.86 (0.46–7.48)	0.38
Food insecurity^f^	6 (8%)	5 (9%)	1 (5%)	1.90 (0.21–17.34)	0.57
Unmet transportation needs^c^	1 (1%)	1 (2%)	0 (0%)	1.14 (0.01–112.28)	0.96
Alcohol misuse^g^	23 (31%)	19 (35%)	4 (20%)	2.17 (0.64–7.43)	0.22
Smoking status^b^					
Never	50 (65%)	38 (67%)	12 (60%)	Reference	
Former	17 (22%)	11 (19%)	6 (30%)	0.58 (0.18–1.90)	0.37
Current	10 (13%)	8 (14%)	2 (10%)	1.26 (0.24–6.78)	0.79

^a^ Missing factor in 9 participants (8 very likely and 1 likely to choose home-based self-collection for screening).

^b^ Missing factor in 1 participant (1 very likely to choose home-based self-collection for screening).

^c^ Missing factor in 2 participants (2 very likely to choose home-based self-collection for screening).

^d^ Missing factor in 5 participants (5 very likely to choose home-based self-collection for screening).

^e^ Missing factor in 3 participants (2 very likely and 1 likely to choose home-based self-collection for screening).

^f^ Missing factor in 3 participants (3 very likely to choose home-based self-collection for screening).

^g^ Missing factor in 4 participants (4 very likely to choose home-based self-collection for screening).

^h^ Missing factor in 1 participant (1 likely to choose home-based self-collection for screening).

CI, confidence interval; OR, odds ratio; SD, standard deviation.

#### Associations between survey responses and likelihood of choosing home-based self-collection

Study subjects had significantly higher odds of reporting that they would be “very likely” to choose self-collection if offered by their health care provider in the future if they also reported that using the Evalyn® brush was very easy (compared with easy, OR: 3.01 [95% CI 1.02–8.88]; *p* = 0.046), if they strongly agreed that using the Evalyn® brush would allow for potential cervical cancer screening by HPV testing (compared with agreed, OR: 5.49 [95% CI 1.82–16.62]; *p* = 0.003), if they strongly agreed that self-collection could improve cervical cancer screening rates (compared with agreed, OR: 4.41 [95% CI 1.50–12.98]; *p* = 0.007), and if they found using the Evalyn® brush to be completely acceptable (compared with acceptable, OR: 7.07 [95% CI 1.96–25.44]; *p* = 0.003) (Table 3). There were no significant associations between odds of being “very likely” to choose self-collection in the future and reported comfort level preself-collection, ease of understanding the instructions, or confidence in correct self-collection.

**Table 3.** Association Between Survey Responses and Likelihood of Choosing Home-Based Self-Collection for Screening Among HPV Self-Collection Study Participants

	Total (*N* = 78)	Very likely (*N* = 58)	Likely (*N* = 20)	OR (95% CI)	*p*-Value
“What was your comfort level before you collected the sample?”					
Very uncomfortable	10 (13%)	9 (16%)	1 (5%)	Reference	
Uncomfortable	4 (5%)	1 (2%)	3 (15%)	0.04 (0.002–0.79)	0.035
Comfortable	29 (37%)	21 (36%)	8 (40%)	0.29 (0.03–2.69)	0.28
Very Comfortable	35 (45%)	27 (47%)	8 (40%)	0.38 (0.04–3.42)	0.38
“Understanding the written instructions for using the Evalyn® brush was _______.”					
Easy	25 (32%)	18 (31%)	7 (35%)	Reference	
Very easy	53 (68%)	40 (69%)	13 (65%)	1.20 (0.41–3.50)	0.74
“Using the Evalyn® brush to collect a sample by yourself for potential cervical cancer screening was _______.”					
Difficult	1 (1%)	1 (2%)	0 (0%)	2.39 (0.02–252.08)	0.71
Easy	21 (27%)	12 (21%)	9 (45%)	Reference	
Very easy	56 (72%)	45 (78%)	11 (55%)	3.01 (1.02–8.88)	0.046
“How confident are you that you collected the sample correctly?”					
Unconfident	4 (5%)	4 (7%)	0 (0%)	5.07 (0.18–141.15)	0.34
Confident	42 (54%)	27 (47%)	15 (75%)	Reference	
Very confident	32 (41%)	27 (47%)	5 (25%)	2.82 (0.92–8.66)	0.071
“I understand that collecting the sample using the Evalyn® brush would allow for potential cervical cancer screening by HPV testing.”^a^					
Strongly disagree	1 (1%)	1 (2%)	0 (0%)	2.85 (0.03–290.44)	0.66
Agree	25 (33%)	13 (22%)	12 (63%)	Reference	
Strongly agree	51 (66%)	44 (76%)	7 (37%)	5.49 (1.82–16.62)	0.003
“Allowing patients to collect their own sample will improve potential cervical cancer screening rates.”					
Strongly disagree	1 (1%)	1 (2%)	0 (0%)	2.89 (0.03–302.65)	0.65
Agree	23 (30%)	12 (21%)	11 (55%)	Reference	
Strongly agree	54 (69%)	45 (78%)	9 (45%)	4.41 (1.50–12.98)	0.007
“Overall, how acceptable was it for you to collect the sample using the Evalyn® brush?”					
Acceptable	13 (17%)	5 (9%)	8 (40%)	Reference	
Completely acceptable	65 (83%)	53 (91%)	12 (60%)	7.07 (1.96–25.44)	0.003

^a^ Missing survey response in 1 participant (1 likely to choose home-based self-collection for screening).

#### Association between sociodemographic factors and overall acceptability of self-collection device use

No significant associations were observed between the multiple sociodemographic variables included in the analysis and strength of overall acceptability (completely acceptable vs. acceptable) of using the self-collection device (Table 4).

**Table 4.** Association Between Sociodemographic Factors and Overall Acceptability of Self-Collection Device Use Among HPV Self-Collection Study Participants

	Total (*N* = 81)	Completely acceptable (*N* = 66)	Acceptable (*N* = 15)	OR (95% CI)	*p*-Value
Age	42.6 (12.3)	41.8 (12.7)	45.9 (10.2)	0.97 (0.93–1.02)	0.25
Race/ethnicity					
White	74 (91%)	62 (94%)	12 (80%)	Reference	
Hispanic or Latino	4 (5%)	3 (5%)	1 (7%)	0.58 (0.06–6.06)	0.65
Other/choose not to disclose	3 (4%)	1 (2%)	2 (13%)	0.10 (0.01–1.15)	0.065
Body mass index^a^					
Normal/underweight	28 (40%)	25 (42%)	3 (27%)	Reference	
Overweight	18 (26%)	15 (25%)	3 (27%)	0.60 (0.11–3.36)	0.56
Obese	24 (34%)	19 (32%)	5 (46%)	0.46 (0.10–2.15)	0.32
Rural-Urban Continuum Codes					
Metro	67 (83%)	56 (85%)	11 (73%)	Reference	
Nonmetro	14 (17%)	10 (15%)	4 (27%)	0.49 (0.13–1.85)	0.29
Physical activity^b^					
Sufficiently active	29 (37%)	22 (34%)	7 (50%)	Reference	
Insufficiently active	43 (54%)	38 (59%)	5 (36%)	2.42 (0.69–8.54)	0.17
Inactive	7 (9%)	5 (8%)	2 (14%)	0.80 (0.13–5.05)	0.81
Stress concern present^c^	34 (44%)	29 (45%)	5 (38%)	1.29 (0.38–4.36)	0.68
Social connections^d^					
Socially isolated	24 (32%)	20 (32%)	4 (31%)	Reference	
Moderately isolated	18 (24%)	15 (24%)	3 (23%)	1.00 (0.19–5.15)	>0.99
Moderately integrated	19 (25%)	17 (27%)	2 (15%)	1.70 (0.28–10.45)	0.57
Socially integrated	14 (19%)	10 (16%)	4 (31%)	0.50 (0.10–2.43)	0.39
At risk of intimate partner violence^e^	5 (7%)	4 (6%)	1 (8%)	0.73 (0.08–7.19)	0.79
Financial resource strain^f^					
Low risk	51 (65%)	42 (66%)	9 (64%)	Reference	
Medium risk	10 (13%)	8 (13%)	2 (14%)	0.86 (0.16–4.73)	0.86
High risk	17 (22%)	14 (22%)	3 (21%)	1.00 (0.24–4.22)	>0.99
Food insecurity^g^	7 (9%)	5 (8%)	2 (14%)	0.52 (0.09–2.99)	0.46
Unmet transportation needs^f^	1 (1%)	1 (2%)	0 (0%)	0.66 (0.01–60.94)	0.86
Alcohol misuse^e^	23 (30%)	20 (31%)	3 (25%)	1.36 (0.33–5.58)	0.67
Smoking status^h^					
Never	50 (63%)	42 (64%)	8 (57%)	Reference	
Former	19 (24%)	15 (23%)	4 (29%)	0.71 (0.19–2.72)	0.62
Current	11 (14%)	9 (14%)	2 (14%)	0.86 (0.16–4.73)	0.86

^a^ Missing factor in 11 participants (7 completely acceptable and 4 acceptable regarding use of the Evalyn® brush).

^b^ Missing factor in 2 participants (1 completely acceptable and 1 acceptable regarding use of the Evalyn® brush).

^c^ Missing factor in 3 participants (1 completely acceptable and 2 acceptable regarding use of the Evalyn® brush).

^d^ Missing factor in 6 participants (4 completely acceptable and 2 acceptable regarding use of the Evalyn® brush).

^e^ Missing factor in 5 participants (2 completely acceptable and 3 acceptable regarding use of the Evalyn® brush).

^f^ Missing factor in 3 participants (2 completely acceptable and 1 acceptable regarding use of the Evalyn® brush).

^g^ Missing factor in 4 participants (3 completely acceptable and 1 acceptable regarding use of the Evalyn® brush).

^h^ Missing factor in 1 participant (1 acceptable regarding use of the Evalyn® brush).

#### Associations between survey responses and overall acceptability of self-collection device use

Study subjects had significantly higher odds of reporting that using the Evalyn® brush to self-collect the vaginal sample was “completely acceptable” if they also reported that understanding the written instructions for using the Evalyn® brush was very easy (compared with easy, OR: 13.60 [95% CI 3.39–54.60]; *p* < 0.001), that using the Evalyn® brush was very easy (compared with easy, OR: 12.11 [95% CI 13.37–43.57]; *p* < 0.001), if they strongly agreed that using the Evalyn® brush would allow for potential cervical cancer screening by HPV testing (compared with agreed, OR: 5.97 [95% CI 1.69–21.13]; *p* = 0.006), if they strongly agreed that self-collection could improve cervical cancer screening rates (compared with agreed, OR: 7.75 [95% CI 2.20–27.29]; *p* = 0.001), and if they endorsed being very likely to choose self-collection in the future (compared with likely, OR: 6.62 [95% CI 1.88–23.26]; *p* = 0.003) (Table 5). There were no significant associations between reported comfort level preself-collection or confidence in correct self-collection and the odds of describing Evalyn® brush use as “completely acceptable” compared with “acceptable.”

**Table 5.** Association between Survey Responses and Overall Acceptability of Self-Collection Device Use Among HPV Self-Collection Study Participants

	Total (*N* = 81)	Completely acceptable (*N* = 66)	Acceptable (*N* = 15)	OR (95% CI)	*p*-Value
“What was your comfort level before you collected the sample?”^a^					
Very uncomfortable	10 (13%)	8 (12%)	2 (13%)	Reference	
Uncomfortable	5 (6%)	4 (6%)	1 (7%)	1.00 (0.07–14.64)	>0.99
Comfortable	30 (38%)	20 (31%)	10 (67%)	0.50 (0.09–2.80)	0.43
Very comfortable	35 (44%)	33 (51%)	2 (13%)	4.13 (0.50–33.91)	0.19
“Understanding the written instructions for using the Evalyn® brush was _______.”					
Easy	27 (33%)	15 (23%)	12 (80%)	Reference	
Very easy	54 (67%)	51 (77%)	3 (20%)	13.60 (3.39–54.60)	<0.001
“Using the Evalyn® brush to collect a sample by yourself for potential cervical cancer screening was _______.”					
Difficult	1 (1%)	1 (2%)	0 (0%)	2.78 (0.03–256.10)	0.66
Easy	22 (27%)	11 (17%)	11 (73%)	Reference	
Very easy	58 (72%)	54 (82%)	4 (27%)	12.11 (3.37–43.57)	<0.001
“How confident are you that you collected the sample correctly?”					
Unconfident	6 (7%)	4 (6%)	2 (13%)	Reference	
Confident	42 (52%)	31 (47%)	11 (73%)	1.41 (0.23–8.80)	0.71
Very confident	33 (41%)	31 (47%)	2 (13%)	7.75 (0.84–71.31)	0.071
“I understand that collecting the sample using the Evalyn® brush would allow for potential cervical cancer screening by HPV testing.”^b^					
Strongly disagree	1 (1%)	0 (0%)	1 (7%)	0.18 (0.002–17.93)	0.47
Agree	26 (33%)	17 (26%)	9 (64%)	Reference	
Strongly agree	53 (66%)	49 (74%)	4 (29%)	5.97 (1.69–21.13)	0.006
“Allowing patients to collect their own sample will improve potential cervical cancer screening rates.”					
Strongly disagree	1 (1%)	0 (0%)	1 (7%)	0.23 (0.002–22.35)	0.53
Agree	25 (31%)	15 (23%)	10 (67%)	Reference	
Strongly agree	55 (68%)	51 (77%)	4 (27%)	7.75 (2.20–27.29)	0.001
“In the future, if your healthcare provider offered the option to use the Evalyn® brush for potential cervical cancer screening at home, how likely would you be to choose that option?”^c^					
Unlikely	1 (1%)	0 (0%)	1 (7%)	0.23 (0.002–22.85)	0.53
Likely	20 (25%)	12 (19%)	8 (57%)	Reference	
Very likely	58 (73%)	53 (82%)	5 (36%)	6.62 (1.88–23.26)	0.003

^a^ Missing survey response in 1 participant (1 completely acceptable regarding use of the Evalyn® brush).

^b^ Missing survey response in 1 participant (1 acceptable regarding use of the Evalyn® brush).

^c^ Missing survey response in 2 participants (1 completely acceptable and 1 acceptable regarding use of the Evalyn® brush).

## Discussion

Among our study population, we observed high levels of understanding the printed directions and ease in using the self-collection device, along with high confidence in having correctly self-collected the vaginal specimen. Study subjects agreed that self-collection could improve cervical cancer screening rates. They reported a high likelihood of choosing self-collection at home if it was offered in the future, and there was consensus that using the Evalyn® brush was acceptable or completely acceptable.

The sociodemographic factors we assessed were not associated with the strength of likelihood of choosing self-collection at home in the future, nor did they predict the strength of overall acceptability. These are promising findings in that characteristics previously reported as being associated with lower rates of traditional speculum-exam cervical cancer screening, such as obesity,^23^ less physical activity,^24^ lack of social support,^25^ and substance use disorders,^26^ were not barriers to interest in and acceptability of self-collection in our surveyed population. We theorize that aversion to speculum examination is common and outweighs any differences in sociodemographic characteristics that might otherwise impact interest in and acceptability of self-collection for cervical cancer screening.

Survey respondents reporting the greatest ease with use of the Evalyn® brush, the strongest agreement with understanding how the Evalyn® brush works for screening and the strongest agreement with the potential for self-collection to increase cervical cancer screening rates, along with those who found using the brush the most acceptable, were found to be most likely to choose self-collection in the future. Similarly, those who found the written instruction for self-collection the easiest to understand, who had the strongest agreement with understanding how the Evalyn® brush works for screening and the strongest agreement with the potential for self-collection to increase cervical cancer screening rates, along with the greatest likelihood of choosing self-collection in the future, were found to have the greatest odds of finding the Evalyn® brush use to be completely acceptable.

Our results are consistent with findings from a Danish study that was also set in a colposcopy clinic and used the Evalyn® brush, with 98% of subjects reporting that the instructions for use were very easy or fairly easy to understand and with 96% describing the test as suitable for use.^27^ Similar results to ours were also noted in two safety-net clinics in Miami in which women (*n* = 120) who completed self-collection of a vaginal specimen in clinic reported that the self-collection device was easy to use (97%), that they felt they had collected the specimen correctly (96%), that they felt comfortable using it in clinic (92%) and would use it again (97%).^17^ In a U.S. nationwide survey of screening-eligible women, 73% reported that they were “probably willing” or “definitely willing” to participate in HPV self-collection at home. These study subjects had not conducted a self-collection, which may have contributed to their concern about correctly obtaining a specimen (51%) but they represented sociodemographics seen in our population with mostly White, overweight/obese, urban/metro, and never smokers respondents.^28^

The priority population for self-collection is un/underscreened people to increase screening access and health equity, whereas we surveyed colposcopy clinic attendees. This limitation was necessary to ensure adequate HPV prevalence for the self-collection device validation component of the study. Another limitation is lack of diversity in our population with mostly White women living in a metro area, and inability to pull educational level from the medical record as a sociodemographic that might impact acceptability of self-collection.

Strengths of our study include surveying women who completed vaginal self-collection for HPV testing in a clinic setting which aligns with the recent FDA approval. As well, to our knowledge, the TFA generic questionnaire has not been previously adapted to measure acceptability constructs of self-collection. The study was conducted in a higher resource setting but one in which community screening rates are comparable with national rates. Thus, we believe it is important to study acceptability and interest in future use of self-collection in this less-studied population.

## Conclusion

Among our study population, regardless of sociodemographic factors, there was great interest in future use of home-based self-collection and almost universal acceptability of using the Evalyn® brush for self-collection in clinic. The recent FDA approval of self-collection in a health care setting provides an opportunity to expand access to screening for those with barriers to a speculum exam. Health care systems should be encouraged to implement this novel screening option.

## Abbreviations Used

BMI: Body Mass Index
CI: Confidence Interval
FDA: U.S. Food and Drug Administration
HPV: Human Papillomavirus
OR: Odds Ratio
TFA: Theoretical Framework of Acceptability

## References

[1] Bray F, Laversanne M, Sung H, et al. Global cancer statistics 2022: GLOBOCAN estimates of incidence and mortality worldwide for 36 cancers in 185 countries. CA Cancer J Clin 2024;74(3):229–263; doi: 10.3322/caac.21834 38572751

[2] Siegel RL, Giaquinto AN, Jemal A. Cancer statistics, 2024. CA Cancer J Clin 2024;74(1):12–49; doi: 10.3322/caac.21820 38230766

[3] National Cancer Institute. Cancer Trends Progress Report. Bethesda, MD; 2024. Available from: https://progressreport.cancer.gov [Last accessed: September 5, 2024].

[4] Serrano B, Ibanez R, Robles C, et al. Worldwide use of HPV self-sampling for cervical cancer screening. Prev Med 2022;154:106900; doi: 10.1016/j.ypmed.2021.106900 34861338

[5] U.S. Food and Drug Administration. FDA Roundup: May 17, 2024. 2024. Available from: https://www.fda.gov/news-events/press-announcements/fda-roundup-may-17-2024#:∼:text=On%20Thursday%2C%20the%20FDA%20granted, or%20after%20platinum%2Dbased%20chemotherapy [Last accessed: July 10, 2024].

[6] Arbyn M, Smith SB, Temin S, et al.; Collaboration on Self-Sampling and HPV Testing. Detecting cervical precancer and reaching underscreened women by using HPV testing on self samples: Updated meta-analyses. Bmj 2018;363:k4823; doi: 10.1136/bmj.k4823 30518635 PMC6278587

[7] Arbyn M, Castle PE, Schiffman M, et al. Meta-analysis of agreement/concordance statistics in studies comparing self- vs clinician-collected samples for HPV testing in cervical cancer screening. Int J Cancer 2022;151(2):308–312; doi: 10.1002/ijc.33967 35179777

[8] Horner-Johnson W, Dobbertin K, Andresen EM, et al. Breast and cervical cancer screening disparities associated with disability severity. Womens Health Issues 2014;24(1):e147–e153; doi: 10.1016/j.whi.2013.10.009 24439941

[9] Amy NK, Aalborg A, Lyons P, et al. Barriers to routine gynecological cancer screening for White and African-American obese women. Int J Obes (Lond) 2006;30(1):147–155; doi: 10.1038/sj.ijo.0803105 16231037

[10] Alcala HE, Mitchell E, Keim-Malpass J. Adverse childhood experiences and cervical cancer screening. J Womens Health (Larchmt) 2017;26(1):58–63; doi: 10.1089/jwh.2016.5823 27500413

[11] Saunders CL, Massou E, Waller J, et al. Cervical screening attendance and cervical cancer risk among women who have sex with women. J Med Screen 2021;28(3):349–356; doi: 10.1177/0969141320987271 33476213 PMC8366122

[12] Weyers S, Garland SM, Cruickshank M, et al. Cervical cancer prevention in transgender men: A review. BJOG 2021;128(5):822–826; doi: 10.1111/1471-0528.16503 32931650

[13] Murphy KA, Stone EM, Presskreischer R, et al. Cancer screening among adults with and without serious mental illness: A mixed methods study. Med Care 2021;59(4):327–333; doi: 10.1097/MLR.0000000000001499 33704103 PMC7952680

[14] Afsah YR, Kaneko N. Barriers to cervical cancer screening faced by immigrant Muslim women: A systematic scoping review. BMC Public Health 2023;23(1):2375; doi: 10.1186/s12889-023-17309-9 38037019 PMC10687813

[15] Vanderpool RC, Jones MG, Stradtman LR, et al. Self-collecting a cervico-vaginal specimen for cervical cancer screening: An exploratory study of acceptability among medically underserved women in rural Appalachia. Gynecol Oncol 2014;132(0 1):S21–S5; doi: 10.1016/j.ygyno.2013.10.008 24125753 PMC4753797

[16] Sewali B, Okuyemi KS, Askhir A, et al. Cervical cancer screening with clinic-based Pap test versus home HPV test among Somali immigrant women in Minnesota: A pilot randomized controlled trial. Cancer Med 2015;4(4):620–631; doi: 10.1002/cam4.429 25653188 PMC4402076

[17] Ilangovan K, Kobetz E, Koru-Sengul T, et al. Acceptability and feasibility of human papilloma virus self-sampling for cervical cancer screening. J Womens Health (Larchmt) 2016;25(9):944–951; doi: 10.1089/jwh.2015.5469 26890012 PMC5311459

[18] Crosby RA, Hagensee ME, Fisher R, et al. Self-collected vaginal swabs for HPV screening: An exploratory study of rural Black Mississippi women. Prev Med Rep 2017;7:227–231; doi: 10.1016/j.pmedr.2016.12.014 28879068 PMC5575437

[19] Anderson C, Breithaupt L, Des Marais A, et al. Acceptability and ease of use of mailed HPV self-collection among infrequently screened women in North Carolina. Sex Transm Infect 2018;94(2):131–137; doi: 10.1136/sextrans-2017-053235 28866635 PMC5831249

[20] MacLaughlin KL, Jacobson RM, Radecki Breitkopf C, et al. Trends over time in pap and Pap-HPV cotesting for cervical cancer screening. J Womens Health (Larchmt) 2019;28(2):244–249; doi: 10.1089/jwh.2018.7380 30614380 PMC6390646

[21] Sekhon M, Cartwright M, Francis JJ. Development of a theory-informed questionnaire to assess the acceptability of healthcare interventions. BMC Health Serv Res 2022;22(1):279; doi: 10.1186/s12913-022-07577-3 35232455 PMC8887649

[22] Sekhon M, Cartwright M, Francis JJ. Acceptability of healthcare interventions: An overview of reviews and development of a theoretical framework. BMC Health Serv Res 2017;17(1):88; doi: 10.1186/s12913-017-2031-8 28126032 PMC5267473

[23] Sand FL, Urbute A, Ring LL, et al. The influence of overweight and obesity on participation in cervical cancer screening: A systematic review and meta-analysis. Prev Med 2023;172:107519; doi: 10.1016/j.ypmed.2023.107519 37080502

[24] Richard A, Rohrmann S, Schmid SM, et al. Lifestyle and health-related predictors of cervical cancer screening attendance in a Swiss population-based study. Cancer Epidemiol 2015;39(6):870–876; doi: 10.1016/j.canep.2015.09.009 26651449

[25] Baeker Bispo J, Lee H, Jemal A, et al. Associations of social support, living arrangements, and residential stability with cancer screening in the United States. Cancer Causes Control 2025;36(2):157–169; doi: 10.1007/s10552-024-01913-0 39422870

[26] Eastment MC, Gupta A, James J, et al. Cervical cancer screening, abnormal results, and follow-up in women with substance use-related diagnoses. Subst Abus 2022;43(1):925–931; doi: 10.1080/08897077.2021.2010257 35289732 PMC9632608

[27] Ornskov D, Jochumsen K, Steiner PH, et al. Clinical performance and acceptability of self-collected vaginal and urine samples compared with clinician-taken cervical samples for HPV testing among women referred for colposcopy. A cross-sectional study. BMJ Open 2021;11(3):e041512; doi: 10.1136/bmjopen-2020-041512 PMC7939007 33674367

[28] Bishop E, Katz ML, Reiter PL. Acceptability of human papillomavirus self-sampling among a national sample of women in the United States. Biores Open Access 2019;8(1):65–73; doi: 10.1089/biores.2018.0040 31057989 PMC6497327

